# ApoER2 expression increases Aβ production while decreasing Amyloid Precursor Protein (APP) endocytosis: Possible role in the partitioning of APP into lipid rafts and in the regulation of γ-secretase activity

**DOI:** 10.1186/1750-1326-2-14

**Published:** 2007-07-09

**Authors:** Rodrigo A Fuentealba, Maria Ines Barría, Jiyeon Lee, Judy Cam, Claudia Araya, Claudia A Escudero, Nibaldo C Inestrosa, Francisca C Bronfman, Guojun Bu, Maria-Paz Marzolo

**Affiliations:** 1 FONDAP Center for Cell Regulation and Pathology (CRCP), Departamento de Biología Celular y Molecular, Facultad de Ciencias Biológicas, Pontificia Universidad Católica de Chile, Santiago, Chile; 2 Departments of Pediatrics and Cell Biology & Physiology, Washington University School of Medicine, St. Louis, Missouri 63110, USA; 3 FONDAP Center for Cell Regulation and Pathology (CRCP), Departamento de Ciencias Fisiológicas, Facultad de Ciencias Biológicas, Pontificia Universidad Católica de Chile, Santiago, Chile

## Abstract

**Background:**

The generation of the amyloid-β peptide (Aβ) through the proteolytic processing of the amyloid precursor protein (APP) is a central event in the pathogenesis of Alzheimer's disease (AD). Recent studies highlight APP endocytosis and localization to lipid rafts as important events favoring amyloidogenic processing. However, the precise mechanisms underlying these events are poorly understood. ApoER2 is a member of the low density lipoprotein receptor (LDL-R) family exhibiting slow endocytosis rate and a significant association with lipid rafts. Despite the important neurophysiological roles described for ApoER2, little is known regarding how ApoER2 regulates APP trafficking and processing.

**Results:**

Here, we demonstrate that ApoER2 physically interacts and co-localizes with APP. Remarkably, we found that ApoER2 increases cell surface APP levels and APP association with lipid rafts. The increase of cell surface APP requires the presence of ApoER2 cytoplasmic domain and is a result of decreased APP internalization rate. Unexpectedly, ApoER2 expression correlated with a significant increase in Aβ production and reduced levels of APP-CTFs. The increased Aβ production was dependent on the integrity of the NPxY endocytosis motif of ApoER2. We also found that expression of ApoER2 increased APP association with lipid rafts and increased γ-secretase activity, both of which might contribute to increased Aβ production.

**Conclusion:**

These findings show that ApoER2 negatively affects APP internalization. However, ApoER2 expression stimulates Aβ production by shifting the proportion of APP from the non-rafts to the raft membrane domains, thereby promoting β-secretase and γ-secretase mediated amyloidogenic processing and also by incrementing the activity of γ-secretase.

## Background

One of the pathological hallmarks of Alzheimer's disease (AD) is the presence of extracellular deposits of the amyloid beta (Aβ) protein [1]. The Aβ peptide, usually ranging from 40 to 43 amino acids in length, derives from the proteolytic processing of the amyloid precursor protein (APP) and has a central role in AD pathology. Aβ peptide has well-established neurotoxic effects when aggregated into oligomeric and fibrillar states, usually seeded by the amyloid prone Aβ_42 _species [2,3] and is also able to interfere with synaptic function, a condition that probably commits neurons to cell death [4-6].

Several cell biology studies on APP metabolism have determined that this membrane protein undergoes two well-compartmentalized processing routes, the amyloidogenic and the non-amyloidogenic [7]. In the amyloidogenic pathway, association of APP to detergent resistant membrane microdomains enriched in cholesterol and glycosphingolipids, also termed lipid rafts, would facilitate the sequential proteolysis of APP by the BACE enzyme (β-secretase) and the γ-secretase complex, generating CTF-β and Aβ plus the signaling related AICD (APP intracellular domain) [8-11]. This is in contrast to APP processing mediated by α-secretase, an enzyme mostly localized at the cell surface and excluded from lipid rafts, whose activity precludes Aβ formation by cutting APP in the middle of the Aβ sequence [12,13]. Several lines of evidence suggest that part of the amyloidogenic processing of APP occurs in the endocytic pathway. Therefore, it has been proposed that internalization of APP increases the production of Aβ [14,15]. The tyrosine based endocytosis motif present in the cytoplasmic domain of APP resembles those found in the endocytic receptors low density lipoprotein receptor (LDL-R) and transferrin receptor (TfR), and mediates the rapid internalization of APP through a clathrin mediated-process in coated pits [16,17]. Mutation of the tetrapeptide YENP within the APP endocytosis motif GYENPTY clearly decreases Aβ generation from cell surface APP [18,19]. However, a strict requirement of endocytosis for amyloid formation is still a matter of debate, since endocytosis blockage by expression of a mutant form of dynamin or by directly co-patching BACE and APP at the cell surface still allowed Aβ production [20,21]. Therefore, it is likely that different pools of the secretase complexes regulating Aβ production are present both at the plasma membrane and within the endocytic pathway.

The LDL-R family of lipoprotein receptors is currently composed of 10 members with a diverse array of ligands with different functions, ranging from cellular cholesterol uptake in the liver to cell specification and neuronal positioning during embryogenesis [22]. Several lines of evidence support a role for these receptors in the pathogenesis of AD, including the participation of one of its ligands, the ε4 isoform of apolipoprotein E as major risk factor for AD [23,24]. LDL-R related proteins (LRPs) share many modular and common structural motifs and usually possess at least one NPxY motif in their relatively short cytoplasmic tail. This motif is critically required for receptor interaction with adaptors proteins and for internalization, with the exception of LRP1, where the endocytosis motif is a YATL [25]. It has been recently demonstrated that several LRPs family members modulate APP processing by affecting different aspects of APP trafficking. For example, LRP1 increases APP endocytosis and Aβ production [26-28], while LRP1B retains APP at the cell surface, preventing it from undergoing amyloidogenic processing [29]. On the other hand, the intracellular trafficking protein SorLA protects cells from generating Aβ by reducing APP trafficking to the cell surface, thereby preventing it from entering the endocytic/amyloidogenic route [30]. Additionally, a particular alternatively spliced isoform of rodent ApoER2 has been demonstrated to interact with APP and decrease Aβ generation upon binding the APP and ApoER2 ligands F-spondin and reelin, respectively [31,32]. Therefore, regulated subcellular localization of APP by LDL-R family members modulates Aβ production by altering APP distribution and interaction with specific secretases (eg. α-secretases or β-secretases).

Recent experimental and clinical evidence suggest that increased plasma cholesterol levels are an important risk factor for AD [33,34] Cholesterol lowering drugs decrease Aβ levels and plaque formation *in vivo *and clinical studies suggest that cholesterol lowering drugs decrease the risk of AD [35-38]. However, data showing the opposite role for cholesterol had also been published (for a review see [39]), illustrating that this is a still a controversial issue. Cholesterol reducing drugs increase sAPPα secretion with a concomitant decrease in Aβ formation, which might occur by shifting APP localization from lipid rafts to α-secretase-containing regions [13,21] and/or decreasing the activity of the amyloidogenic enzymes. In fact, cholesterol depletion disrupts β- and γ-secretase association to lipid rafts and causes a decrease in Aβ production [8-10] indicating that lipid rafts are relevant sites for amyloidogenic processing of APP.

ApoER2 is a member of the LDL-R family that is highly expressed in the brain [40]. This receptor has a slower endocytosis rate compared to LRP1 [25], although its internalization is clathrin-dependent [41]. A significant fraction of ApoER2 is also found in lipid rafts, but the disruption of these membrane microdomains does not affect its endocytic activity [41]. Based on its slow endocytic rate, it was initially proposed that the physiological functions of ApoER2 might be in signal transduction pathways rather than the endocytosis of lipoproteins and other ligands [25]. It is currently known that ApoER2 participates in the reelin signaling pathway during neuronal development [42-44], and recent evidence indicates that it also participates in novel neuronal functions such as maturation of NMDA receptor composition in the hippocampus [45] and the regulation of long term potentiation [46,47]. Importantly, it has been determined that the ApoER2 ligand reelin is found in neuritic plaques of transgenic mice overexpressing APP [48-50], suggesting a possible association with AD. It has been shown that one splice variant of the mouse ApoER2 receptor regulates the processing of APP [31,51]. However, there are no studies addressing whether full length human ApoER2 modulates APP trafficking and consequently its processing. Here, we demonstrate that ApoER2 interacts with APP and increases APP cell surface levels while decreasing APP internalization. However, the increased cell surface APP correlated with increased Aβ production. In addition, ApoER2 expression induced an increase in APP association with lipid rafts and a decrease APP-CTFs levels, which in part could be explained by the increase in γ-secretase activity found in ApoER2 expressing cells. Overall our results indicate that ApoER2 might constitute a novel modulator of APP processing by affecting its endocytic trafficking and the proportion of APP present in lipid rafts, as well as the activity of the γ-secretase complex.

## Results

### ApoER2 cytoplasmic domain is important for increasing APP at the cell surface

**Figure 1:**
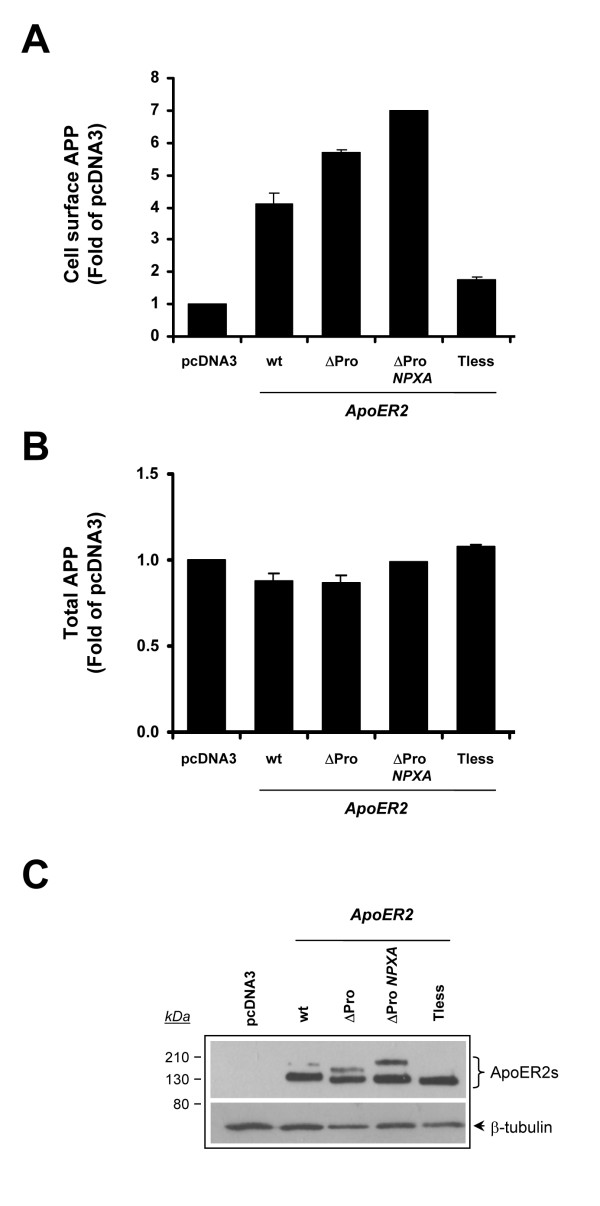
**ApoER2 increases cell surface APP levels but not total APP**. Cell surface APP (*A*) and total cellular APP (*B*) were assessed by FACS analysis. LRP1-null CHO cells stably transfected with pcDNA3 vector, ApoER2, ApoER2ΔPro, ApoER2ΔPro-NPXA, or ApoER2-Tless were treated with or without saponin and then labeled with anti-APP antibody. Primary antibody was detected with PE-conjugated goat anti-mouse IgG. Values are the average of triplicate determinations. *Error bars *indicate S.E. *C*, lysates were prepared from CHO-pcDNA3 and ApoER2-expressing cells and equal amounts of protein were subjected to 10% SDS-PAGE. Blots were probed with anti-HA and anti-β-tubulin antibodies.

The internalization of cell surface APP represents one of the major pathways for generation and subsequent release of Aβ into the extracellular space. In order to evaluate a potential role for ApoER2 in APP metabolism, we analyzed the effect of human ApoER2 expression on APP subcellular distribution in CHO cells lacking endogenous LRP1, which is known to regulate APP processing and Aβ levels [26-28,52]. We determined cell surface APP levels by flow immunocytometry in cells expressing a series of ApoER2 variants (Fig. 1A). Initially, we detected increased cell surface APP levels in CHO-ApoER2 compared to CHO-pcDNA3 cells. This increase is not due to an increase in total APP, as determined in parallel experiments with cells permeabilized with saponin (Fig 1B). *apoER2 *gene produces several splice isoforms, including an isoform lacking a 59-amino acid insert coding for a proline-rich domain within the cytoplasmic region [53]. We then asked whether this domain is important for the effect of ApoER2 on APP cell surface levels. In fact, the expression of the receptor isoform devoid of the proline-rich insert, ApoER2ΔPro, produced an even higher increase in cell surface APP than the observed with full length ApoER2 expression (Fig 1B and 1C). These results demonstrate that both ApoER2 splice variants affect steady state, cell surface APP levels and suggest that the proline-rich domain partially inhibits the mechanism(s) responsible for the increment of cell surface APP.

In order to determine whether the molecular determinants for the ApoER2-induced effect on APP trafficking are located in the cytoplasmic domain of the receptor, we analyzed APP cell surface levels in CHO cells expressing the internalization mutants of ApoER2 [41]. We have previously determined that truncation of the entire intracellular domain of ApoER2 (tail less version of ApoER2) promotes its accumulation within intracellular compartments in CHO cells (mostly endoplasmic reticulum) thereby preventing its access to the cell surface. However, a single point mutation (Y/A) in the NPxY sequence of the ApoER2ΔPro isoform abolishes its endocytosis, thereby increasing cell surface ApoER2ΔPro levels. Importantly, both mutated receptors still retain its ability to bind RAP, indicating that alterations imparted to the receptors do not affect their folding and ligand binding [41]. Therefore, we analyzed APP cell-surface levels in CHO-ApoER2-Tailess and CHO-ApoER2ΔPro-NPxA cells. Deletion of the ApoER2 C-terminal domain completely abolished the ApoER2-induced effect on APP subcellular localization. In contrast, the mutation of the NPxY motif within ApoER2ΔPro construct caused an additional slight increase in APP cell surface levels, suggesting that reduced endocytosis of ApoER2 promotes APP accumulation at the cell surface (Fig. 1A, B). These results demonstrate that ApoER2 regulates APP subcellular location and that the cytoplasmic domain of the receptor is important to accomplish this increase in cell surface levels of APP.

### ApoER2 interacts with APP695 independently of its cytoplasmic domain

**Figure 2:**
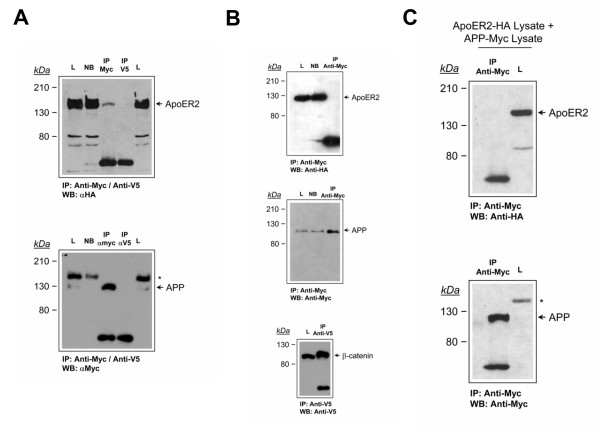
**ApoER2 interacts with APP in N2a cells**. (*A*), co-immunoprecipitation of ApoER2 with APP. Cell extracts were prepared from ApoER2-expressing cells transiently transfected with APP695-Myc. Extracts were immunoprecipitated with anti-Myc or with the unrelated, anti-V5 antibody and probed for ApoER2 and APP with the anti-HA and anti-Myc antibodies, respectively. *L*, input lysate. NB, not bound. (*B*), cell extracts were prepared from untransfected ApoER2-expressing cells and processed as in *A*. anti-Myc antibody does not immunoprecipitate ApoER2 by itself. *Middle panel*, anti-Myc antibody do immunoprecipitate APP695Myc in single transfected cells. *Lower panel*, anti-V5 antibody immunoprecipitation idoneity was confirmed by β-catenin-V5 precipitation from transfected N2a cells. (*C*), independent lysates from ApoER2-expressing cells and from APP695-Myc-transfected cells were combined and the co-immunoprecipitation experiment was continued with the anti-Myc antibody as in *A*. ApoER2-HA and APP695-Myc requires an *in vivo *context for interaction. Remnants of ApoER2 detection after stripping and reblot are indicated by an asterisk.

Given the functional interaction between APP and ApoER2, we then performed co-immunoprecipitation experiments in N2a cells in order to test whether these proteins do interact. We choose N2a cells because this cell type lacks detectable expression for any of the LDL-R family members [41]. We lysed N2a cells expressing both APP695-Myc and ApoER2-HA and immunoprecipitated APP with anti-Myc antibody. Western blot of immunoprecipitates probed with anti-HA indicates that ApoER2 co-precipitates with APP (Fig. 2A, *Upper panel*). Blots were stripped and re-blotted with anti-Myc to demonstrate that APP695 was effectively immunoprecipitated (Fig. 2A, *Lower panel*). Neither anti-Myc nor anti-HA immunoreactive bands were seen in lysates immunoprecipitated with the non-related immunoglobulin anti-V5 antibody, which efficiently immunoprecipitates β-catenin-V5 (Fig. 2A and Fig. 2B, *Lower panel*), demonstrating that true immune complexes were isolated. As a control for specificity of the anti-Myc antibody, similar experiments were performed in ApoER2-expressing cells not transfected with APP695-Myc. The anti-Myc antibody does not immunoprecipitate ApoER2-HA (Fig. 2B, *Upper panel*) but do immunoprecipitates APP695-Myc (Fig. 2B, *middle panel*) in single transfected cells, demonstrating that ApoER2 precipitation with anti-Myc requires the APP protein. For testing an *in vivo *requirement for this interaction, independent lysates from ApoER2-HA and APP695-Myc expressing cells were incubated together and then immunoprecipitated with anti-Myc. No ApoER2-HA was detected in the APP695-Myc-containing immunoprecipitate (Fig. 2C). These results strongly suggest that APP and ApoER2 directly interact in N2a cells.

**Figure 3:**
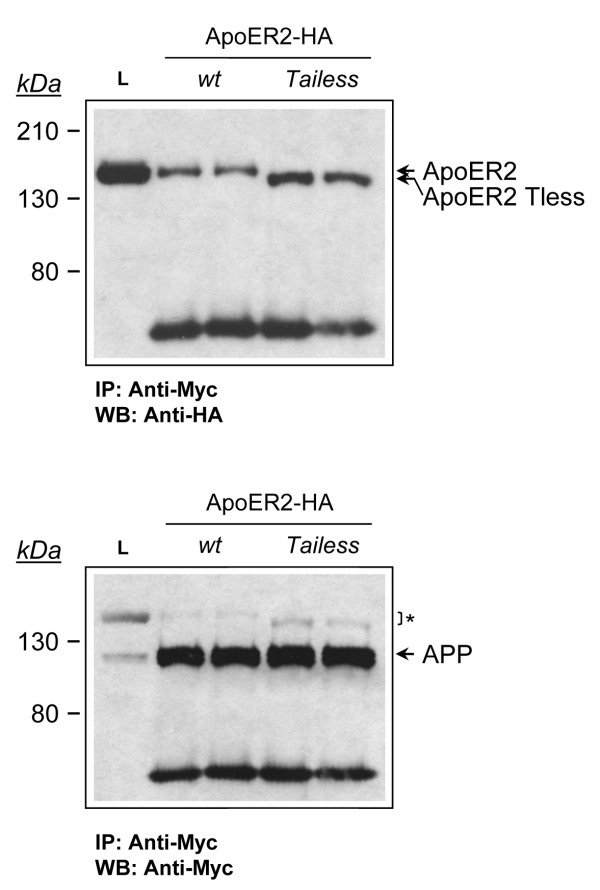
**ApoER2 and APP interact independently of the cytosolic domain of ApoER2**. Cell extracts were prepared from ApoER2- and ApoER2 Tailess-expressing N2a cells transiently transfected with APP695-Myc. Extracts were immunoprecipitated with anti-Myc and probed for ApoER2 and for APP with the anti-HA and anti-Myc antibodies, respectively. Remnants of ApoER2 detection after stripping and reblot are indicated by an asterisk.

To determine whether this interaction occurs through the intracellular domain of both proteins, we analyzed co-immunoprecipitation of APP695-Myc with ApoER2 in N2a cells transiently transfected with APP695-Myc and stably expressing HA-tagged wild type ApoER2- or ApoER2-Tailess, which in the case of N2a cells is able at least in part to get the plasma membrane [41]. When the immunoprecipitates were blotted with anti-HA, we found that both wild type ApoER2 and ApoER2-Tless co-immunoprecipitated with APP695 (Fig. 3), indicating that the intracellular domain of the receptor is dispensable for the interaction with APP.

### ApoER2 co-localize with APP695 at the plasma membrane and within early endocytic vesicles

**Figure 4:**
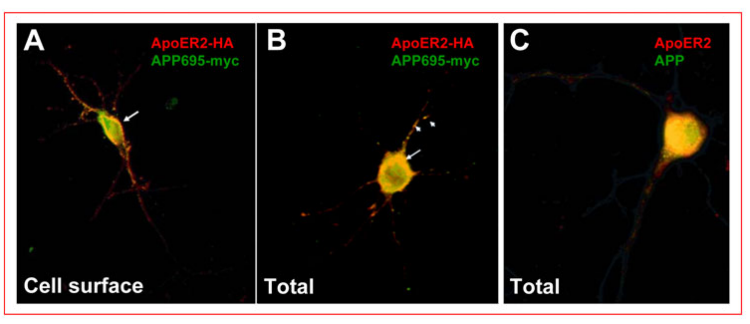
**ApoER2 and APP colocalization in hippocampal neurons**. Primary cortical neurons were co-transfected with APP-Myc and ApoER2-HA and the receptors immunodetected in non-permeabilized (A) and permeabilized (B) cells using anti-Myc and anti-HA antibodies (*C*) Hippocampal primary neurons were permeabilized and labeled for total APP and ApoER2 with anti-APP(6E10) and C-terminal anti-ApoER2, respectively. APP695-Myc and ApoER2-HA partially co-localize at the soma surface (*A*, arrows) and within neuronal processes (arrowheads). A strong co-localization of APP and ApoER2 in a perinuclear region of permeabilized cells for both overexpressed (*B*) and endogenous levels (*C*) was evidenced.

**Figure 5:**
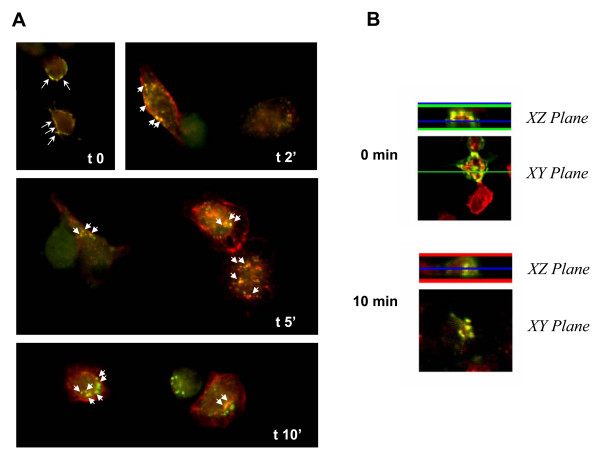
**ApoER2 and APP co-localize at the cell surface and within internalization vesicles of N2a cells**. N2a cells were transiently transfected with ApoER2-HA and APP695-Myc and surface-immunolabeled with anti-HA and anti-Myc antibodies for 60 min at 4°C. The cells were then shifted to 37°C for varying periods of time, to allow internalization to occur. (*A*) Prior to warming (t0), ApoER2 and APP immunofluorescence was confirmed at the cell membrane (*arrows*). Partial co-immunolocalization (*arrowheads*) was detected as early as 2 min after endocytosis in punctuate structures near the cell membrane. By 10 minutes, most of the immunoreactivity was confined to deeper internalized vesicles, as visualized by confocal XZ-plane analysis (*B*) at t0 and t 10 min.

The finding that ApoER2 interacts with APP and modulates APP cell surface levels led us to determine whether both proteins co-localize in cortical and hippocampal neurons, where both proteins are naturally expressed. Both in transfected cortical neurons expressing APP695-Myc and ApoER2-HA, as well as in hippocampal neurons where endogenous proteins were immunodetected, APP colocalizes with ApoER2 at the cell surface (Fig. 4A, C). In addition, we observed co-localization in a perinuclear region resembling recycling compartments and/or the *trans*-Golgi network (Fig. 4B). As ApoER2 and APP are internalized through a clathrin-mediated process, we asked whether both proteins could actually be co-internalized from the cell surface. APP and ApoER2 co-internalization was assessed using an immuno-labeling assay in living cells. N2a cells expressing APP695-Myc and ApoER2-HA were incubated at 4°C with anti-Myc and anti-HA antibodies. After washing, the cells were then warmed to 37°C for different periods of time to initiate endocytosis, followed by fixation, permeabilization and staining with Alexa-conjugated secondary antibodies. In cells that were labeled and processed at 4°C, we found that both proteins co-localized, analyzing the samples by both conventional (Fig. 5A, 0 min) and confocal (Fig. 5B) microscopy. The XZ-plane analysis shows a co-localization at the cell surface in co-transfected cells. As early as 2 minutes after warming, most of the ApoER2 immunofluorescence was still confined to the cell surface but a small fraction of ApoER2 was observed within intracellular punctuate structures that also contain APP immunoreactivity (Fig. 5A, 2 min, *arrowheads*). The intracellular co-localization of both proteins was more evident with longer incubation times. The XZ-plane analysis at 10 min demonstrates co-localization within endocytic vesicles. Only background was observed when using antibodies against intracellular proteins or secondary antibodies only in control experiments (data not shown). These results provide direct evidence that APP and ApoER2 localize to the same subcellular compartments upon endocytosis and suggest that increased APP cell surface levels might be the result of a direct interaction of ApoER2 and APP at the cell surface.

### ApoER2 diminishes APP695 endocytosis and increases Aβ production

**Figure 6:**
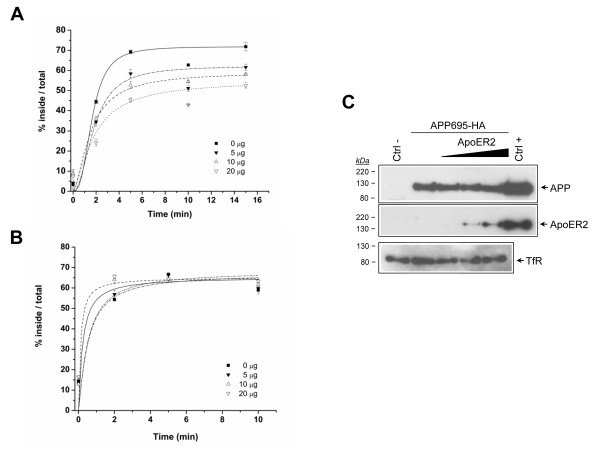
**ApoER2 expression specifically decreases APP695HA endocytosis**. Dose-response effect of ApoER2 expression on APP695HA (*A*) and diferric transferrin (*B*) endocytosis. CHO-LRP null cells transiently transfected with APP695HA alone (solid square) or along with 5 μg (solid inverted triangle), 10 μg (open triangle) or 20 μg (open inverted triangle) of an ApoER2 coding plasmid were incubated with 1 nM ^125^I-labeled-anti-HA IgG or 5 nM ^125^I-labeled-diFeTf for 1 h at 4°C and then shifted to 37°C for the indicated times. The percent of ligand internalized at each time point is equal to the amount of ligand internalized divided by the total cell-associated ligand (see "Materials and Methods") Values are the mean average of triplicate determinations with S.E. indicated by *error bars*. (*C*) Lysates were prepared from similarly transfected cells and equal amounts of protein were subjected to 10% SDS-PAGE. Blots were probed with anti-HA, anti-ApoER2, anti-actin or anti-transferrin receptor (TfR) antibodies. ApoER2 expression decreases APP695 but not transferrin receptor endocytosis rate.

The ApoER2-induced increase in steady state cell surface APP levels could be caused either by a decrease in APP internalization or by increased recycling of APP to the cell surface. In order to analyze this point, we analyzed APP endocytosis in CHO LRP1-null cells transiently transfected with APP695HA along with increasing amounts of ApoER2. Cell surface APP was bound to ^125^I-antiHA at 4°C. After 1 h the cells were washed and warmed for different times, and cell surface and internalized pools of APP were determined as the radioactivity in acid labile- and cell associated-fractions, respectively [25]. Results were plotted as % inside-to-total at each time point. The endocytosis rates are defined considering the ligand internalization half-times, corresponding to the time required for a 50% internalization to occur [25,54]. APP695 was rapidly internalized with an estimated half-time of 2 minutes (Fig. 6A, Additional File 1: Table I). Interestingly, the expression of ApoER2 caused a dose-dependent increase in the half-time of APP695 internalization and a decrease in the extent of APP695 internalization (Additional File 1: Table I). To confirm that this effect was due to ApoER2 expression, cells from parallel experiments were lysed and proteins were analyzed for steady state ApoER2 and APP expression by Western blot (Fig. 6C). Increased ApoER2 expression was detected where decreased APP endocytosis is observed, suggesting that the effect directly depends on ApoER2 expression. As both ApoER2 and APP can be endocytosed by a clathrin mediated processes [17-19,41] we reasoned that the effect of ApoER2 might be due to a simple competition for limited, ubiquitous endocytic factors. To rule out this possibility, the same experiments were performed, but iodinated diferric transferrin instead of anti-HA was now used as a ligand for endocytosis kinetic determinations. The transferrin receptor possesses a very fast initial endocytic rate (half-time of Tfr internalization < 2 min) [55] (Fig. 6B). Expression of even the highest levels of ApoER2 (Fig. 6C) did not alter the internalization kinetics of the transferrin receptor, demonstrating that the expression of ApoER2 specifically alters the efficiency of APP endocytosis (Additional file 1: Table I). These results strongly suggest that the increased cell surface APP levels observed in ApoER2-expressing cells are due to a specific effect in APP endocytosis.

**Figure 7:**
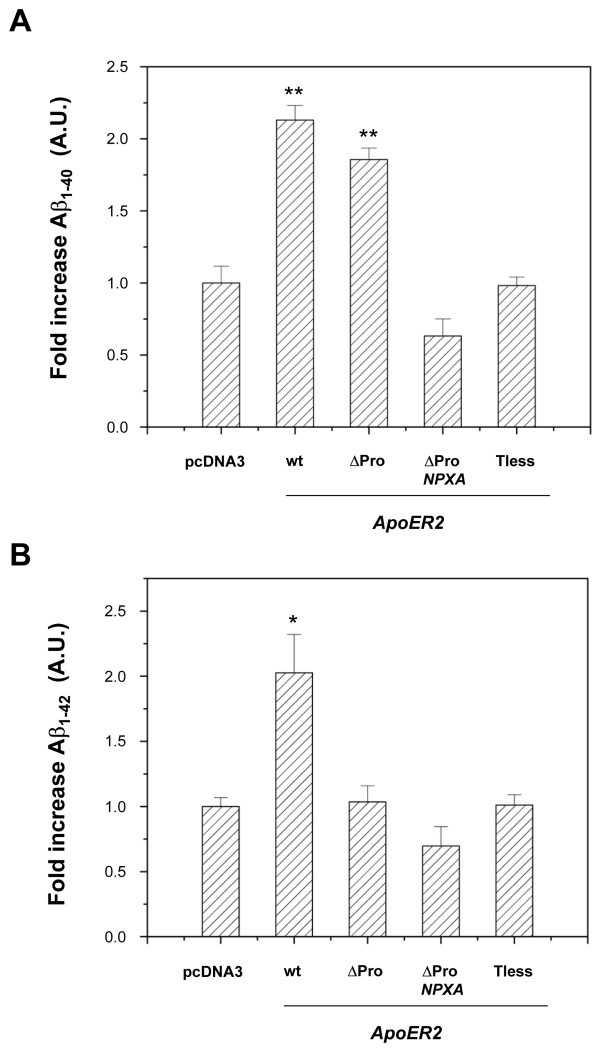
**Increased endogenous Aβ secretion in LRP1-null, ApoER2-expressing CHO cells**. LRP1-null CHO cells stably transfected with pcDNA3 vector, ApoER2, ApoER2ΔPro, ApoER2ΔPro-NPXA, or ApoER2-Tless encoding plasmids were incubated in low serum media. After 48 h, media was analyzed for Aβ levels by ELISA. (*A*) Aβ40 and (*B*) Aβ42 values were normalized to the amount of cellular protein in the corresponding cell extracts and expressed as fold increase over pcDNA3-expressing cells. Values are the mean average of triplicate determinations with S.E. indicated by *error bars*.

A growing body of evidence has established members of the LDL-R family as key regulators of the amyloidogenic processing of APP. During the course of this study, findings published by the work of Hoe et al [32] also demonstrated that ApoER2 interacts with APP and that this interaction can be regulated by the extracellular APP ligand F-spondin, causing a net decrease in Aβ production but increased APP and ApoER2-CTF levels. However, the ApoER2 isoform used in that work corresponds to a mouse variant lacking 3 ligand binding repeats in the extracellular domain. Therefore, we asked whether human full length ApoER2, as well as several ApoER2 variants that alter APP subcellular distribution (Fig. 1), might also modify Aβ secretion in CHO cells lacking LRP1. Conditioned media from cells expressing different ApoER2 isoforms and mutants were collected and analyzed for Aβ_40 _and Aβ_42 _by ELISA. Unexpectedly, Aβ_40 _levels were significantly increased in cells expressing both ApoER2 and ApoER2ΔPro isoforms (Fig. 7A). The increase in Aβ_40 _levels was dependent on the integrity of the NPxY motif, indicating that the ApoER2 endocytosis and/or the recruitment of cytosolic proteins were required for this processing to occur. To gain further insight into a positive correlation between increased cell surface APP and increased Aβ production, we analyzed Aβ levels in CHO cells expressing the ApoER2-Tailess mutant, which does not reach cell surface [41]. Cells expressing this construct do not show an increase in cell surface APP (Fig. 1). Consistently, the truncation of the cytoplasmic domain of ApoER2 abolishes the ApoER2-induced increase in Aβ_40 _levels (Fig. 7A). To test whether the more pathologically relevant Aβ_42 _was also modulated by ApoER2, we similarly determined Aβ_42 _levels by ELISA in conditioned media from ApoER2-expressing cells. Aβ_42 _levels paralleled those of Aβ_40 _in most of the cell lines analyzed. However, Aβ_42 _was increased only in cells expressing the full length receptor but not in cells expressing ApoER2ΔPro isoforms (Fig. 7B). Collectively, these results demonstrate that ApoER2 modulates trafficking and processing of APP towards the amyloidogenic pathway at the cell surface and in an isoform dependent way, a new scenario for regulated APP processing induced by an LDL-R family member.

### ApoER2 increases APP695 association to lipid rafts but decreases APP-CTF

**Figure 8:**
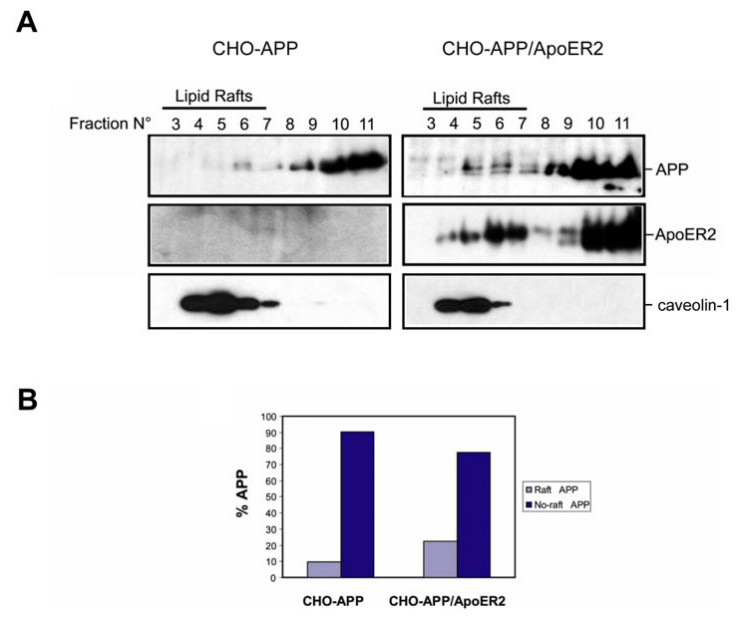
**ApoER2 increases full length APP association to lipid rafts in LRP1-null CHO cells**. (*A*) Increased association of APP to lipid rafts in ApoER2-expressing cells. LRP1-null CHO cells were transiently transfected with pcDNA3 (*control*) and APP695-Myc (*APP*) or APP695-Myc along with ApoER2-HA, treated with 10 μM DAPT for 16 h and lipid rafts were isolated in Lubrol 0.5% – containing lysis buffer in a sucrose gradient. After concentration of sucrose gradients fractions, proteins were subjected to 6% SDS-PAGE, blotted and probed with anti-APP and anti-ApoER2 antibodies. To demonstrate that ApoER2 expression does not disrupts lipid rafts formation in APP expressing cells, 1/16 sucrose gradients fractions were subjected to 12.5% SDS-PAGE and probed with anti-caveolin antibody. ApoER2 increases lipid raft association of full length APP. (*B*), densitometric analysis of a representative Western blot experiment of (*A*).

Recent retrospective clinical studies [35,36] as well as several *in vitro *and *in vivo *evidence [37,38,56] support the hypothesis that increased cellular cholesterol levels increase APP metabolism through the amyloidogenic pathway and that decreasing cholesterol levels increases non-amyloidogenic processing [13]. Cellular cholesterol effects on the amyloidogenic processing might occur by facilitating association of APP with either the BACE or the γ-secretase complex, which reside in detergent insoluble membrane microdomains enriched in cholesterol, called lipid rafts [8-11]. ApoER2 is a receptor that associates to lipid rafts, and this characteristic is not related to its internalization pathway [41]. Therefore it is possible that the increase in Aβ levels we determined could be explained by an ApoER2-induced shift of APP to lipid rafts, where APP proteolytic processing occurs. To test this possibility, we prepared Lubrol-insoluble membrane domains from APP or APP-ApoER2 expressing CHO LRP1-null cells at 4°C and detected APP in lipid rafts isolated by sucrose density gradient. As previously described for Lubrol lysed cells [8,10], most of the cellular APP was present in the non-rafts fractions (9 to 11); however ApoER2 expression led to a 1.2 fold increase (measured densitometrically) in the proportion of APP associated to lipid rafts (not shown). Interestingly when γ-secretase activity was inhibited with DAPT [57] we found that APP was better detected in lipid rafts fractions. The lipid raft pool of APP was further incremented when ApoER2 was co-expressed (Fig. 8A). Densitometric analysis of Western blots indicates that ApoER2 expression causes a 2.2 fold increase in APP distribution into lipid rafts (Fig. 8B). Detection of the receptor in the same membranes, with an anti-ApoER2 antibody, confirms the receptor expression only in transfected cells and demonstrates that fully-glycosylated, mature ApoER2 and full length APP coexist at the same buoyancy membrane fractions. The gradients sucrose profile and caveolin-1 distribution for control and ApoER2 co-transfected cells were similar, confirming that protein overexpression does not alter the integrity of lipid rafts (Fig. 8A). Considering that ApoER2 and APP can be co-immunoprecipitated (Fig. 2) and that both proteins co-localize, at the plasma membrane and after co-internalization (Fig. 4, Fig. 5) these results strongly suggest that the effect of ApoER2-induced APP raft localization might be direct.

**Figure 9:**
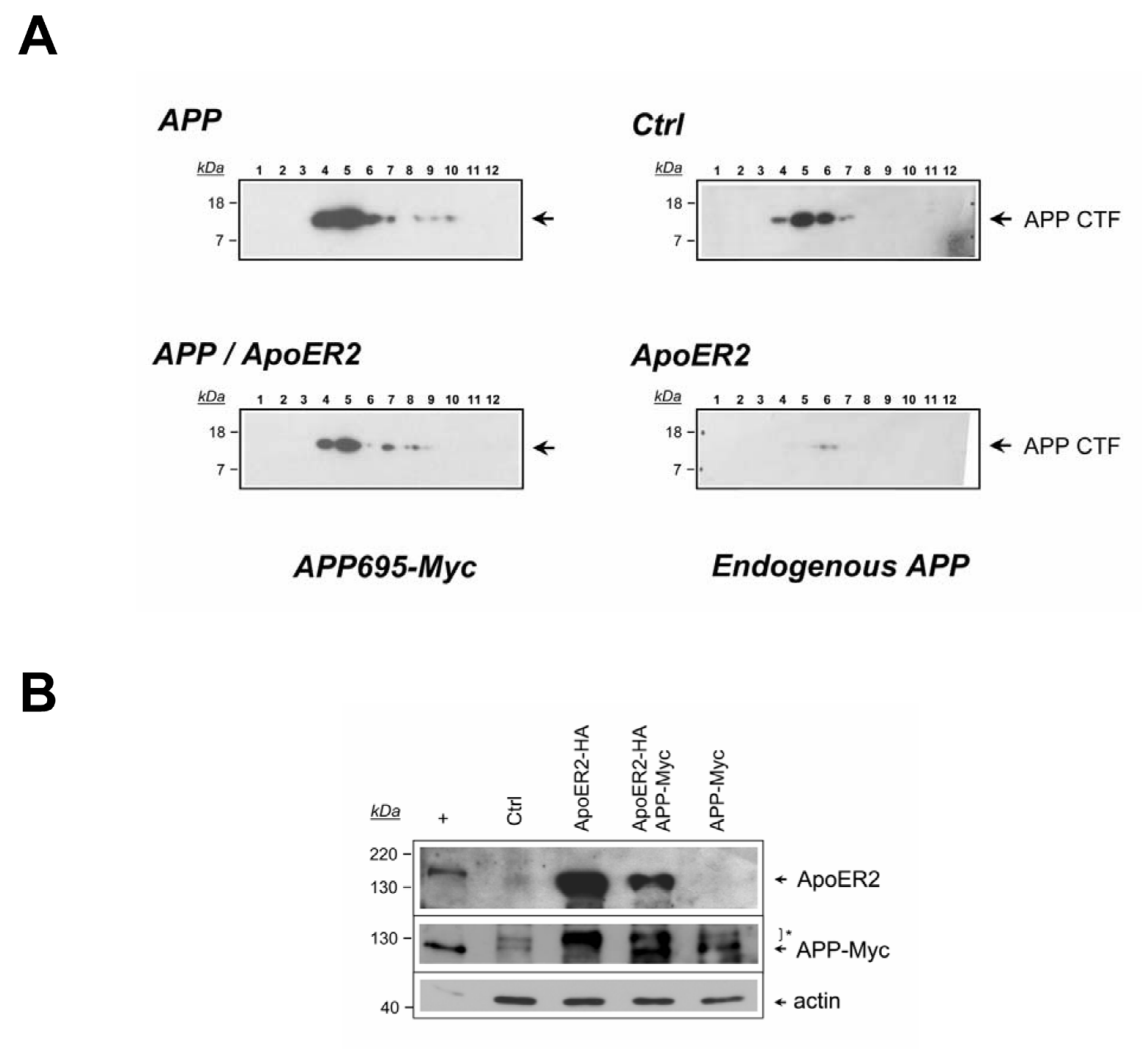
**ApoER2 expression decreases APP-CTFs in DAPT-treated LRP1-null CHO cells**. (*A*) ApoER2 decreases both endogenous hamster APP-CTFs and human APP695-CTFs. LRP1-null CHO cells were transiently transfected with pcDNA (*control*), APP695-Myc (*APP*) or APP695-Myc along with ApoER2-HA (*APP/ApoER2*). Cells were then treated with DAPT 10 μM and protein association to lipid rafts was analyzed as in Figure 8. High sensitivity films were used for endogenous APP-CTFs detection. (*B*), Lubrol-extracted proteins from lysates obtained in (*A*) were subjected to 10% SDS-PAGE. Blots were probed with anti-ApoER2, anti-APP and anti-actin or antibodies. Similar APP protein levels were detected in APP695-Myc and in APP695-Myc + ApoER2-HA transfected cells. Remnants of ApoER2 detection after stripping and reblot are indicated by an asterisk.

To further support the notion of an ApoER2-induced amyloidogenic processing of APP, we asked whether ApoER2 expression modifies APP-CTF levels. It has been previously determined that in addition to the amyloidogenic secretase machinery and a small proportion of APP, lipid rafts also contain APP-CTFs and Aβ [10,58]. Therefore we analyzed the effect of ApoER2 expression in the levels of APP-CTF fragments obtained in the lipid rafts from Lubrol-extracted cells. Under control conditions, APP-CTFs were not detected in cells expressing APP695 (Additional file 2, A left panels). In fact, it has been shown that the detection of APP-CTF fragments critically depends on the inhibition of γ-secretase activity [10,59]. Then, in the presence of DAPT, we were able to detect clearly APP-CTFs (Additional file 2, A right panels). In both cases we detected ApoER2-CTFs but also ApoER2-ICDs, which suggests an incomplete inhibition of γ-secretase activity in the presence of DAPT (Additional file 2B). We then characterized the effect of ApoER2 expression on the processing of endogenous and transfected APP (in the presence of DAPT). Surprisingly, ApoER2 expression significantly decreased the steady state levels of APP-CTFs in CHO LRP1-null cells expressing endogenous (hamster) APP or transfected human APP695 (Fig. 9A). Western blot analyses from total cell lysates denoted similar human APP expression in both conditions (Fig. 9B). Collectively, these results indicate that ApoER2 increases full-length APP association to rafts but diminishes APP-CTF levels, suggesting that ApoER2 regulates APP processing in multiple steps.

**Figure 10:**
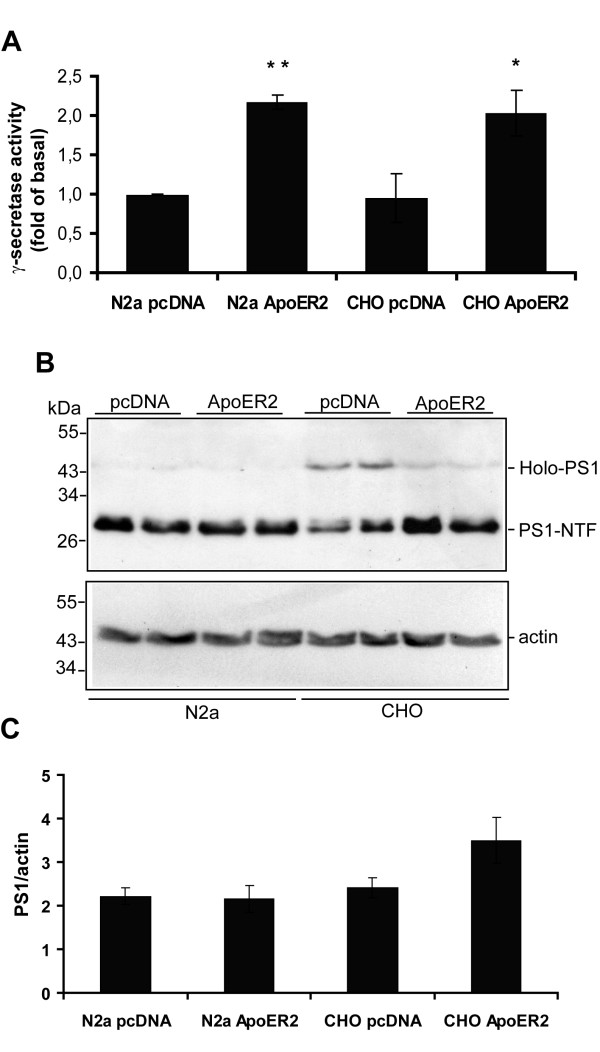
**ApoER2 expression enhances γ-secretase activity**. N2a and CHO LRP1-null cells expressing full length ApoER2 or transfected with pcDNA3 alone were lysed in CHAPSO containing buffer. (*A*), Measurement of γ-secretase activity using a fluorogenic substrate assay, which is based on the secretase-dependent cleavage of a γ-secretase-specific substrate conjugated with a fluorescent molecule. Data represent mean ± S.E.M of three experiments in duplicated, presented as fold values of the basal activity. * P < 0.05, ** P < 0.01. (*B*), 50 μg of protein/lane were subjected to 12% SDS-PAGE and endogenous PS1 levels were determined by Western blot. In N2a cells almost all PS1 is detected as PS1-NTF but in CHO LRP1-null cells a significant amount of Holo-PS1 is also detected. ApoER2 expression increases PS1-NTF and decreases Holo-PS1 compared to control CHO LRP1-null cells. The expression of actin was determined in the same blot as a loading control. (*C*), densitometric analysis of Western blots as in (*B*) confirms that total PS1 levels were slightly increased in CHO-ApoER2 cells compared to the pcDNA3 cells. However, a further increase in the PS1-NTF fraction is clearly observed in the Western blot.

The decreased levels of APP-CTFs and the presence of more Aβ suggest that the basal activity of the amyloidogenic processing enzymes could be enhanced in cells expressing ApoER2. However the fact that the levels of APP-CTF are decreased in the presence of ApoER2 does not necessarily support an increment in β-secretase activity. The increment in Aβ levels could be explained for a more efficient γ-secretase processing on APP-CTFs in the presence of ApoER2 and similar activity of β-secretase. To test this hypothesis we compared γ-secretase activity from CHAPSO-solubilized membrane fractions of N2a and CHO LRP1-null cells expressing vector or ApoER2, by mixing these fractions with an APP-CTF derived fluorescent peptide harboring the γ-secretase cleavage site. It should be noted that CHAPSO protects the integrity of the γ-secretase complex and hence activity and therefore has been widely used to measure γ-secretase activity [60-63]. We found that γ-secretase activity was significantly enhanced in two different cell lines expressing ApoER2, N2a and CHO LRP1-null (Figure 10A). The measured activity was sensitive to the γ-secretase inhibitor DAPT (see methods). In N2a cells, the total amount PS1 was not changed (Figure 10B, C), being almost exclusively in the form of the active PS1-NTF. Interestingly in CHO cells, the total PS1 (Holo-PS1 and PS1-NTF) related to actin was slightly increased in cells expressing ApoER2 but the PS1-NTF levels were clearly increased in CHO ApoER2 (Figure 10B, compare Holo-PS1 with PS1-NTF) suggesting that the expression of the receptor could influence the autocatalytic activation of PS1. Our novel findings show that the activity of γ-secretase is enhanced in cells expressing ApoER2 and this effect could explain, at least in part, the increment in Aβ and the decreased levels of APP-CTFs in the same ApoER2 cells compared to the controls.

## Discussion and Conclusion

In this work we assessed the role of ApoER2 in APP trafficking and processing in cells that do not express LRP1, another receptor of the LDL-R family previously described as a modulator of APP processing [64]. Our findings show that ApoER2 decreased APP internalization rate and increased the amount of APP that partitions into lipid rafts, where Aβ is produced. Interestingly, the expression of ApoER2 also significantly increased γ-secretase activity in two different cell types compared to controls. As a net result of these changes, Aβ levels were significantly increased. The effects that ApoER2 has on APP trafficking and processing might be direct, as ApoER2 and APP co-immunoprecipitate and co-localize at the cell surface of neuronal cells and are also found within the same intracellular vesicles upon internalization. Our results show that ApoER2 increases Aβ production despite elevated cell surface APP levels, due to effects on APP association with lipid rafts and γ-secretase activity. This presents a significant and novel mechanism for regulation of APP metabolism by an LDL-R family member.

The catabolism of APP is a well-compartmentalized process. While the predominant non-amyloidogenic pathway occurs mostly if not exclusively at the cell surface, the less frequent amyloidogenic branch occurs either along the biosynthetic pathway during APP delivery to the cell surface or during APP trafficking to endosomes upon internalization from the cell surface [7]. All the LDL-R family proteins that have been demonstrated to interact with APP and affect its processing modify APP trafficking and change Aβ production by modulating the ratio of cell surface/intracellular APP. For example, increased APP localization to intracellular compartments in cells expressing the endocytic LRP1 receptor promotes Aβ generation [27,28]. On the contrary, expression of the tumor suppressor protein LRP1B, which has a very slow endocytic rate, increases APP levels at the plasma membrane and reduces Aβ production [29]. Another LRP family member, the intracellular sorting related protein SorLA/LR11, interacts with APP in intracellular compartments and modulates its trafficking to the trans-Golgi network, preventing its delivery to the cell surface for subsequent internalization and amyloidogenic processing [30,65,66] and its expression is decreased in AD [30]. Ectopic localization of SorLA/LR11 or LRP1 to the cell surface increases cell surface APP with a concomitant decrease in Aβ production, highlighting that the APP amyloidogenic process is facilitated during the cell surface/endocytosis cycles of APP [28,30]. In a completely opposite scenario, here we demonstrated that human ApoER2 increases APP levels at the cell surface but also increases Aβ levels from endogenous APP in CHO LRP1-null cells. This ApoER2-mediated effect on APP cell surface was also observed with the alternatively spliced ApoER2 isoform lacking the proline rich domain. Interestingly, Aβ42 levels were increased in cells expressing full length ApoER2 but not ApoER2ΔPro, while Aβ40 production was increased in both. It is known that Aβ40 is formed predominantly at the *trans*-golgi network and endosomes, while Aβ42 is produced in earlier intracellular compartments, mainly intermediate compartments and *cis*-Golgi cisternae [7]. Although the overall endocytic properties of ApoER2 and ApoER2ΔPro are virtually identical [41] differences in Aβ42 production might be the result of different APP trafficking routes during the biosynthetic pathway and/or after internalization induced by ApoER2. Alternatively, the association of ApoER2 with adaptor proteins that specifically interact with the proline rich domain, such as PSD-95 or JIP-1/2, might account for these differences [47,67,68]. Further experiments are required to address this hypothesis.

In the course of our experiments, similar findings were published in the work by Hoe et al. [32] showing a physical and functional interaction between ApoER2 and APP695. In that case, however, the secreted protein F-spondin serves as an extracellular link for the interaction of these proteins, which results in increased cell surface APP levels but decreased Aβ levels. Two possible explanations for the discrepancies between these data and those presented here are (i) the different ApoER2 splice variant isoforms used and (ii) the different LRP1 status of the cell lines utilized. In the work by Hoe et al. [32], they utilized a mouse version of ApoER2 lacking the entire exon 5 coding for repeats 4 to 6 in the ligand binding domain, the main alternatively spliced isoform found in mouse [69]. In the present study, we used the full length ApoER2 version containing all 15 exons encoding the extracellular domain [70]. It is worth mentioning that ApoER2 isoforms lacking repeats 4 to 6 affected activated α2 M but not β-VLDL binding to ApoER2 [71,72] suggesting that this domain may play a role in binding APP. Although we can not rule out that F-spondin might also increase human full-length ApoER2 interaction with APP and decrease Aβ secretion, this domain may be important for differential binding to other ligands required for the ApoER2-APP functional interaction to modulate Aβ production. Importantly, two additional isoforms of human ApoER2 lacking repeats number four to six and four to seven in the ligand binding domain are also expressed in the brain [70-72] but nothing is known about the regulation of these isoforms during aging or in AD. On the other hand, in our studies we also used two cell lines that lack LRP1 which increases Aβ production but also directly binds and internalizes Aβ [64]. The CHO LRP1-null cells we used here were previously used for assessing the effect of LRP1 mutants on APP processing [27,28] and in the N2a cells, we did not inmunodetect LRPs or RAP binding activity [41]. Therefore, and in contrast to the experimental system used before [32], in our study, we were able to dissect these possible LRP1 mediated-events from the effect induced by ApoER2 expression alone.

Early biochemical assays utilizing cell surface radioiodination and endocytosis inhibition by potassium depletion highlighted the importance of APP endocytosis in Aβ production [18], and subsequent evidence with APP mutants clearly defined molecular determinants required for clathrin-mediated endocytosis of APP and Aβ production [19,27]. Our results show that ApoER2 decreases endocytosis of APP but surprisingly, increases Aβ production. Recent evidence indicates that APP can also be processed at the cell surface. Endocytosis inhibition by expression of the dominant negative Dynamin-1 protein, DynK44A, increased both Aβ and p3 secretion in HeLa cells [20]. However, the same mutant decreased Aβ secretion in HEK293 stably expressing APP695 [73]. It has been reported that PS1 can be delivered along with Nicastrin to the plasma membrane in a functional complex, where it might proteolyse Notch, Erb4, E-cadherin and APP at the γ-secretase cleavage site [74]. Interestingly, exciting results have been published by the work of Ehehalt et al. [21] showing that, despite APP endocytosis is required for Aβ production, co-patching of BACE and APP at cell surface lipid rafts bypasses the detrimental effect of APP endocytosis inhibition on amyloidogenic processing arguing that cell surface Aβ production can take place in particular conditions within surface lipid rafts. In our work, we demonstrated that ApoER2 increased the amount of APP fractionating into lipid rafts. Therefore, an increased amyloidogenic processing of APP at plasma membrane lipid rafts might explain the effects induced by ApoER2 (i.e. increased association to lipid rafts and decreased endocytosis rate) and reconcile the increased Aβ production and decreased APP internalization we have observed. Consistent with this notion, LRP1 has been recently recognized as a BACE substrate and both LRP1 and BACE interact at the cell surface [75], suggesting that other LDL-R family members, including ApoER2, might also be processed by BACE along with the interacting protein APP. As lipid rafts are also located to intracellular compartments (early endosomes, TGN and recycling vesicles [76]), we can not rule out however that APP that is slowly endocytosed by interaction with ApoER2 expression might routed via clathrin-dependent endocytosis to a lipid raft enriched intracellular compartment for amyloidogenic processing where ApoER2, BACE and the γ-secretase complex have been detected [11,14,41,77-79].

In addition to the increase in APP present in lipid rafts, our results also show a new and interesting effect of ApoER2 expression on the γ-secretase activity. The novel results support the possibility that the increased Aβ levels might also result from increased γ-secretase activity induced by ApoER2 within intracellular compartments. Supporting this model, recently published evidence by Pei and colleagues showed that, β-adrenergic receptor activation induces PS1 endocytosis through a clathrin-mediated process and increases Aβ production [62]. Therefore, an ApoER2-induced delivery of APP to lipid rafts and an increase in γ-secretase activity might also provide the basis of the increased Aβ production by ApoER2 we have observed. Despite our results linking ApoER2 expression with APP-CTFs do not suggest an induction in β-secretase activity, we cannot discard that ApoER2 expression also modifies this proteolytic activity.

Cholesterol positively regulates γ-secretase and Aβ selectively regulates key enzymes for cholesterol metabolism [80]. On the other hand, the raft associated ApoER2 is also a γ-secretase substrate that is proteolytically processed upon apoE binding [31,81]. Therefore we suggest that neuronal ApoER2 would have a physiological role in APP processing and function, which could be functionally connected to the neuronal lipid homeostasis. Deregulation of a concerted APP/ApoER2 processing might play a role in the pathogenesis of Alzheimer's disease.

## Methods

### Cell lines and cell tissue culturing

Parental CHO LRP-1 null cells were grown in F12HAM/10%FBS supplemented with 100 U Penicillin, 1 U Streptomycin and 5 μg/mL Plasmocin. The ApoER2-HA, ApoER2-ΔPro-HA, ApoER2-ΔPro-NPXA-HA and ApoER2Tless-HA-expressing CHO LRP1-null cells have been previously described [41] and were grown in the presence of 400 μg/mL G418. Parental N2a were grown in DMEM/7.5% FBS supplemented with 100 U Penicillin, 1 U Streptomycin and 5 μg/mL Plasmocin. ApoER2-HA and ApoER2-Tless-HA-expressing N2a cells [41] were maintained in growing medium supplemented with 400 μg/mL G418. CHO and N2a cells were transfected using Lipofectamine2000 and different amount of plasmid as noted. For control experiments, total DNA was kept constant by including empty pcDNA3 vector in the transfection mixture. Three h after the addition of the DNA-liposome complexes, the cells were washed with PBS and grown overnight in serum-containing media without antibiotics.

### Determination of cell surface APP levels by FACS in CHO LRP-1 null clones

Cells (4 × 10^6^) were plated and grown overnight in 100 mm Petri dishes. Cells were washed with PBS and incubated at 37°C in 3 mL PBS-EDTA 1 mM for 5 min. After the addition of 3 mL complete medium, cells were mechanically detached and collected (40% or 60% of the total) by centrifugation at 700 × *g *for 5 min in a 15 mL conical tube. The smaller pellet was dissolved in 160 μL PFN (1.5% heat-inactivated FBS, 0.1% NaN_3 _in PBS) and kept on ice. The remaining cells were resuspended in 160 μL PFN-saponin 0.05% and gently mixed at 4°C for 30 min. Permeabilized cells were similarly collected by centrifugation at 4°C for 10 min and resuspended in 160 μL PFN. Permeabilized and non-permeabilized cells were then equally divided to two microcentrifuge tubes, and 50 μL PFN (control samples) or 50 μL PFN containing 50 ng/μL of anti-APP (6E10, Signet, previously used to detect endogenous APP from CHO cells, [29]) were added. After gently rocking the tubes at 4°C for 60 min, cells were washed twice with 800 μL PFN or PFN-saponin 0.05%, respectively and resuspended in 50 μL PFN or 50 μL PFN-saponin 0.05% containing 20 μg/mL PE-conjugated donkey anti-mouse IgG (Molecular Probes), respectively. After 40 min secondary antibody incubation at 4°C, cells were similarly washed and resuspended in 300 μL PFN for FACS determinations in a FACScalibur cytometer (Beckton & Dickinson). Surface and total APP fluorescence was represented as the geometric mean of fluorescence intensity from non-permeabilized and permeabilized cells, respectively, after the subtraction of the corresponding blank controls. Results are plotted as % of pcDNA3 control.

### ApoER2 and APP co-immunoprecipitation in N2a cells

4 × 10^6 ^cells of ApoER2-HA or ApoER2-Tless-HA-expressing N2a cells were grown overnight in 100 mm Petri dishes. Cells were washed twice with PBS and transfected with 30 μg of APP695-Myc plasmid [29] using Lipofectamine2000. 48 h after transfection, the cells were washed twice with PBS and lysed 30 min at 4–10°C in 500 μL Buffer HUNT (20 mM Tris-HCl pH 8.0, 100 mM NaCl, 1 mM EDTA, 1% NP40, 0.5% TX-100, 50 mM NaF) supplemented with 2 mM PMSF and 2× protease inhibitors cocktail. After debris removal by centrifugation, cell lysates were precleared with 20 μL agarose-protein A/G (Pierce) for 30 min at 4°C and then incubated for 2 h at 4°C with 5 μg anti-Myc (clone 9E10, Roche) with gentle rocking. 40 μL agarose-protein A/G (Pierce) was then added, and the mixtures were incubated for 1.5 h at 4°C with gentle rocking. Beads were washed three times with 1 mL Buffer HUNT supplemented with 1 mM PMSF, boiled in 30 μL reducing Laemli Buffer and 7.5% SDS-PAGE was performed. Anti-V5 (Invitrogen) was used as a non-related antibody and mock transfected ApoER2-HA-expressing cells or APP695-Myc only expressing cells were used as controls. For the *in vivo *requirement experiment, parental N2a cells were analogously transfected in a 60 mm plate and equivalent HUNT lysates from APP-transfected and ApoER2-HA-expressing cells were combined and the standard protocol was followed.

### Colocalization analysis in primary rat hippocampal and cortical neurons

Hippocampal and cortical neurons were obtained and cultured as described [82]. Dissociated cells were plated on glass-coverslips coated with 1 mg/ml poly-L-lysine in medium containing 10% horse serum (Invitrogen). After 3 hr, the medium was supplemented with N2 (Invitrogen) [83]. Cells were transiently transfected on day 7 with plasmids encoding APP695-myc and ApoER2-HA using LipofectAMINE 2000. After 18 h, neurons were fixed with 4% PFA and 4% sucrose for 20 min at 37°C, and processed for immunofluorescence as described previously [84]. For cell surface staining, incubation with primary or secondary antibodies was performed before permeabilization with detergents (saponin). Endogenous detection was assessed in hippocampal neurons using anti-APP (6E10, Signet) and anti C-terminal ApoER2 antibodies. Stained cells were analyzed with an inverted microscope (Leica, DM2000) equipped with epifluorescence filters and photographed using a 100× objective. Alternatively, a Zeiss laser scanning confocal microscope was used to collect X-Y sections in 0.45 μm using a 60× objective.

### Co-endocytosis of ApoER2-HA and APP695-Myc in N2a cells

N2a cells (8 × 10^5^) were plated in 35 mm Petri dishes and grown overnight. Cells were washed twice with PBS and transfected with plasmids encoding APP 695-Myc (4.5 μg), ApoER2-HA (1.75 μg) and RAP (1.75 μg). After 24 h, the cells were tripsinized and grown in 6 × 10 mm coverslips for an additional day. The cells were chilled on ice for 30 min, washed twice with ice-cold PBS and incubated with 30 μL Binding Buffer (2% BSA in DMEM) containing 1 μg/mL chicken anti-Myc (AB3252, Chemicon) and 1:30 dilution of an anti-HA hybridoma supernatant (Clone 12CA5) for 90 min at 4°C. After washing away excess antibody with PBS, the cells were incubated with 1 mL pre-warmed Binding Buffer for the indicated times at 37°C. Endocytosis was immediately stopped by quickly washing the cells with ice-cold PBS and incubating in 600 μL PFA 2% for 20 min. The cells were permeabilizaed in PBS/saponin 0.2% for 10 min and nonspecific binding sites were blocked with PBS/gelatin 0.2% for 1 h. A mixture of 2 μg/mL donkey anti-mouse Alexa 488 (Molecular Probes) and donkey anti-chicken Alexa 596 (Molecular Probes) secondary antibodies in PBS/gelatin 0.2% was added for 30 min at 37°C and the covers were mounted with DABCO and analyzed either by indirect immunofluorescence with Leica microscope or by confocal microscopy. As a control, cells transfected with 8 μg of pcDNA3 were similarly processed.

### Preparation and radioiodination of Diferric transferrin and anti-HA

Diferric transferrin (diFeTf) was prepared from apotransferrin (apoTf) as described previously [85] with some modifications. 87.5 μL of 10 mg/mL human apoTf in 0.1 M Hepes-KOH pH 7.4 were combined with 3.5 μL of 5 mM Fe(NTA)_2_. After adding 109 μL of 1 M NaHCO_3_, the mixture was incubated 30 min at 37°C, and full saturation of apoTf with Fe was monitored by an Abs_465/280 _ratio of 0.044. Routinely, diFeTf was obtained at 90% saturation. The anti-HA antibody was purified as previously described and eluted in PBS [29]. 25–50 μg of protein was iodinated in 10 mM sodium phosphate buffer pH 7.5 in a total reaction volume of 100 μL with 1–2 mCi Na [^125^I] by using IODOGEN(Pierce)-treated tubes. After 10 min incubation at room temperature, free iodine was removed by passing the mixture through a PD-10 desalting column (GE healthcare) and iodinated proteins were recovered as one mL eluted fractions. Specific activities of radioligands usually ranged from 30,000 to 50,000 cpm/ng and were stored at 4°C until use.

### Radioligand endocytosis in transfected CHO LRP-1 null cells

4 × 10^6 ^cells were plated in 100 mm Petri dishes and grown overnight. Cells were washed twice with PBS and transfected with a mixture of plasmids containing 20 μg APP695-HA [29], 5 μg RAP and ranging from 5 to 20 μg ApoER2 as noted using Lipofectamine 2000. Total DNA was kept constant by including empty pcDNA3 vector where needed. 24 h after transfection, the cells were split into 15 wells of a single column of five 12-well multiplates for the endocytosis assay, as well as 2 wells for Western blot analysis. After an additional 24 hours of culture, the cells were chilled on ice for 30 min, washed twice with ice cold Binding Buffer (0.6% BSA in F12HAM) and incubated 1 h at 4°C with 500 μL Binding Buffer containing 1 nM ^125^I-anti-HA. After eliminating unbound antibody with two cold Binding Buffer washes, the cells were incubated with 1 mL pre-warmed Binding Buffer and incubated for indicated times at 37°C. Endocytosis was stopped by immediately placing the plate on ice and adding 1 mL cold Stop Strip solution (0.2 M acetic acid, 100 mM NaCl, pH 2.6). Cell surface radioligands were detached from cells twice by incubating the cells in 1 mL Stop Strip for 10 min at 4°C, and internalized radioligands were then recovered by lysing cells in 1 mL Low SDS Buffer (62.5 mM Tris-HCl, 0.2% SDS, 10% glycerol, pH 6.8). Acid labile and endocytosed fractions were counted in a γ-counter and plotted as % internalized-to-total radioactivity ratio versus time. As a specificity control for the antibody, radioactivity measurements were corrected by subtracting the corresponding background radioactivity obtained from similarly processed, control cells. The time at which 50% internalization occurs was referred as the internalization half-time and is inversely related to the endocytosis rate. The same protocol was used for diFeTf endocytosis, except that cells were pre-incubated for 2 h with Binding Buffer at 32°C for maximal recovery of TfR at the cell surface prior to binding of ^125^I-diFeTf, 5 nM.

### Verification of protein expression levels by Western blot

Overnight cultures were scraped in lysis buffer (PBS containing 1% TX-100) supplemented with 1 mM PMSF and a protease inhibitor cocktail for 30 min at 4°C. After nuclei and debris removal by centrifugation, proteins were quantified and 30 μg proteins were subjected to 10% SDS-PAGE and transferred to PVDF membranes. Lysates from gradients were directly used for quantification and Western blotting. In the case of PS1 determination cell extracts were prepared in CHAPSO buffer (50 mM Tris-HCl, 2 mM EDTA, protease inhibitors (Roche), 0.25% CHAPSO pH 6.8). PVDF membranes were blocked with PBS/5% low fat milk and incubated O/N with PBS/5% low fat milk supplemented with the indicated antibody and 0.05% Tween 20 for anti-APP (CT695, Zymed; 1:500), anti-HA (mouse hybridoma supernatant; 1:100), anti-TfR (H68.4, Zymed; 1:1,000), anti-PS1 (1:5,000), [86] anti-actin (MAB1501R, Chemicon; 1:4,000); or 0.1% Tween 20 for anti-ApoER2 antiserum (1:3,000) at 4°C in a roller. After 3 washes, the membranes were incubated 3 h at room temperature in a roller with HRP coupled anti-mouse IgG antibody (Sigma; 1:5,000) or HRP coupled anti-rabbit HRP (Sigma; 1:10,000). After 4 washes, the membranes were incubated with 10 mL enhanced chemiluminescence reagent and low or high sensitivity films were appropriately used. Quantification of bands was performed using the MATRIX (Quantavision) software.

### Aβ40 and Aβ42 determinations

CHO LRP-1 null stable clones (1 × 10^5 ^cells) were plated in 35 mm Petri dishes and grown for 48 h. After washing the cells twice with PBS, the cells were grown in 800 μL F12HAM/1%FBS supplemented with antibiotics and 400 μg/mL G418 for additional 48 h. Conditioned media were collected, centrifuged at 20,000 × *g *for 5 min at 4°C and Aβ40 and Aβ42 concentrations in the cleared conditioned media were determined by sandwich ELISA as previously described [28]. Briefly, serial dilutions of Aβ40 and Aβ42 in F12HAM/1%FBS were prepared for standard curves. 100 μL freshly thawed conditioned media or standard dilutions were subjected to ELISA sandwich using anti-Aβ_1–42 _(21F12) or anti-Aβ_13–28 _(266) antibody-covered plates. After overnight incubation at 4°C, wells were extensively washed with guanidine containing buffer, and biotin-conjugated anti-Aβ_13–28 _(266) antibody or anti-Aβ_1–40 _(2G3) were added for Aβ40 and Aβ42 detection, respectively. After 90 min incubation at 37°C, avidin-conjugated HRP was added for 90 min, and ELISA was developed with TMB substrate in a SoftMax ELISA machine, reading the plate at 650 nm every 2 min. For normalization, total protein extracts were prepared from the same growing plates by scrapping the cells in lysis buffer (TX-100 1% in PBS) supplemented with 1 mM PMSF and a protease inhibitor cocktail. After nuclei and debris removal by centrifugation, proteins were quantified and Aβ levels were normalized to protein content per well.

### Immunodetection of full-length and CTFs of ApoER2 and APP in lipid rafts

4 × 10^6 ^cells were grown overnight in 100 mm Petri dishes. Cells were washed twice with PBS and transfected with a mixture of plasmids containing either 20 μg APP, a mixture of 5 μg RAP and 5 μg ApoER2 or both using Lipofectamine 2000. Mock transfected cells were used as a negative control for endogenous APP detection. 24 h after transfection, the cells were split into two 100 mm Petri dishes and treated overnight with 10 μM DAPT or vehicle. The cells were then chilled on ice for 30 min, washed twice with cold PBS, scraped in Lysis Buffer (25 mM MES pH 6.5, 150 mM NaCl, 0.5% Lubrol WX) supplemented with 2 mM PMSF and a 2× protease inhibitors cocktail and incubated on ice for 1 h with periodic vortexing. Lysates were passed through a G27 needle 5 times and debris was removed by centrifugation at 10,000 × *g *for 3 min at 4°C. 500 μL of lysate were combined with 500 μL of 80% sucrose in MBS (25 mM MES pH 6.5, 150 mM NaCl) and briefly mixed by vortex. Subsequently, 2 mL of 35% sucrose and 2 mL 5% sucrose in MBS were carefully added for discontinuous gradient formation and samples were centrifuged at 130,000 × *g *for 18 h at 4°C in an AH650 Sorvall Rotor. Twelve 400 μL fractions were recovered from top to bottom, and 300 μL were precipitated by the methanol/chloroform method [87]. Total precipitated proteins were subjected to Tris-Tricine SDS-PAGE for ApoER2- and APP-CTF immunodetection or to 6% SDS-PAGE for full length ApoER2/APP immunodetection by Western blot. In some experiments, gradient fractions were subjected to sucrose content determination and caveolin-1 immunodetection as previously described [41].

### γ-Secretase-mediated peptide cleavage assay

γ-Secretase activity was assayed in vitro using an APP-CTF derived intramolecularly quenched fluorescent peptide according to the manufacturers' instructions (Calbiochem) [60,63]. Briefly, cellular membranes from N2a and CHO cell were prepared as described [63] and solubilized in CHAPSO buffer (50 mM Tris-HCl, 2 mM EDTA, protease inhibitors (Roche), 0.25% CHAPSO pH 6.8) followed by incubation at 37°C for different times in 150 μl of assay buffer containing 50 mM Tris-HCl, protease inhibitors (Roche), 2 mM EDTA, 0.25% CHAPSO, pH 6.8 and 8 μM fluorescent APP-CTF derived peptide (Calbiochem). After incubation, the reaction mixture was centrifuged (16,100 × g, 15 min) and the supernatant transferred to a 96 well plate. Fluorescence was measured using a Perkin Elmer Luminescence spectrometer LS50B (excitation/emission at 350/440 nm). Specific γ-secretase activity was determined after subtracting the fluorescence obtained in the presence of DAPT (10 μM). Background fluorescence was calculated by incubating separately 50 μg of CHAPSO-solubilized P2 membranes and 8 μM APP-CTF derived peptide with assay buffer for different times and mixing them just before fluorescence determination.

## Competing interests

The author(s) declare that they have no competing interests.

## Authors' contributions

RAF was a postdoctoral fellow in Dr. Marzolo's lab and carried out most of the experiments described in this article, contributed with the analysis of the data and worked in the figures and manuscript preparation. MIB contributed significantly to the work performing the first observations of APP cell surface levels and Aβ production and also making many stable cell lines used in this study. JL and JC performed the ELISA measurements of Aβ and contributed with their expertise in the result's interpretation. CA and CAE contributed with the γ-secretase activity measurements. NI contributed in the beginning of this project with his expertise in Aβ and with the results interpretation and support. GB and FB significantly contributed conceptually to the design of some experiments and helped to draft the manuscript. MPM is the corresponding author, contributing intellectually with the conception of the project and analysis and interpretation of the data, direction of the personnel and also participated in the manuscript writing. All authors read and approved the final manuscript.

## Supplementary Material

Additional file 1 **Table 1 – ApoER2 expression affects the extent of APP695HA internalization and the half-time of ^125^I-antiHA internalization in a dose dependent manner**. Parameters were calculated from sigmoidal fit of data from Figure 6. Click here for file

Additional file 2 **Figure: Effect of DAPT treatment on APP and ApoER2 CTFs**. LRP1-null CHO cells were transiently transfected with plasmids encoding APP695-Myc or ApoER2-HA. After 24 h, cells were incubated with 10 μM DAPT or vehicle (DMSO) for 16 h and lipid rafts were then isolated in a sucrose gradient. After concentration of the sucrose gradients fractions, proteins were subjected to Tris Tricine-PAGE, blotted and probed with anti-APP (*A*) and anti-ApoER2 (*B*) antibodies. DAPT treatment improves APP-CTFs detection. However apparently there is still remaining activity that explains the presence of ApoER2-ICDs, resulting from processing of ApoER2-CTFs. Click here for file
