# Supplementary material for: ApoER2 expression increases Aβ production while decreasing Amyloid Precursor Protein (APP) endocytosis: Possible role in the partitioning of APP into lipid rafts and in the regulation of γ-secretase activity
Source: Mol Neurodegener. 2007 Jul 9;2:14. doi: 10.1186/1750-1326-2-14 (PMC1939850; doi:10.1186/1750-1326-2-14)
Supplement: Additional file 1 — Table 1 – ApoER2 expression affects the extent of APP695HA internalization and the half-time of 125I-antiHA internalization in a dose dependent manner. Parameters were calculated from sigmoidal fit of data from Figure 6. [file 1750-1326-2-14-S1.pdf]

Table I

ApoER2 expression affects the extent of APP695HA internalization and the half-time of ^{125}I-antiHA internalization in a dose dependent manner*

μg ApoER2HA	**maximal internalization (% of total)**	**^{125}I-antiHA internalization ($t_{1/2}$) (min)**
0	71.7	2.3
5	61.7	3.6
10	57.6	4.6
20	52.4	9.2

* parameters were calculated from sigmoidal fit of data from Figure 6.
