# Supplementary figures and images for: ApoER2 expression increases Aβ production while decreasing Amyloid Precursor Protein (APP) endocytosis: Possible role in the partitioning of APP into lipid rafts and in the regulation of γ-secretase activity
Source: Mol Neurodegener. 2007 Jul 9;2:14. doi: 10.1186/1750-1326-2-14 (PMC1939850; doi:10.1186/1750-1326-2-14)

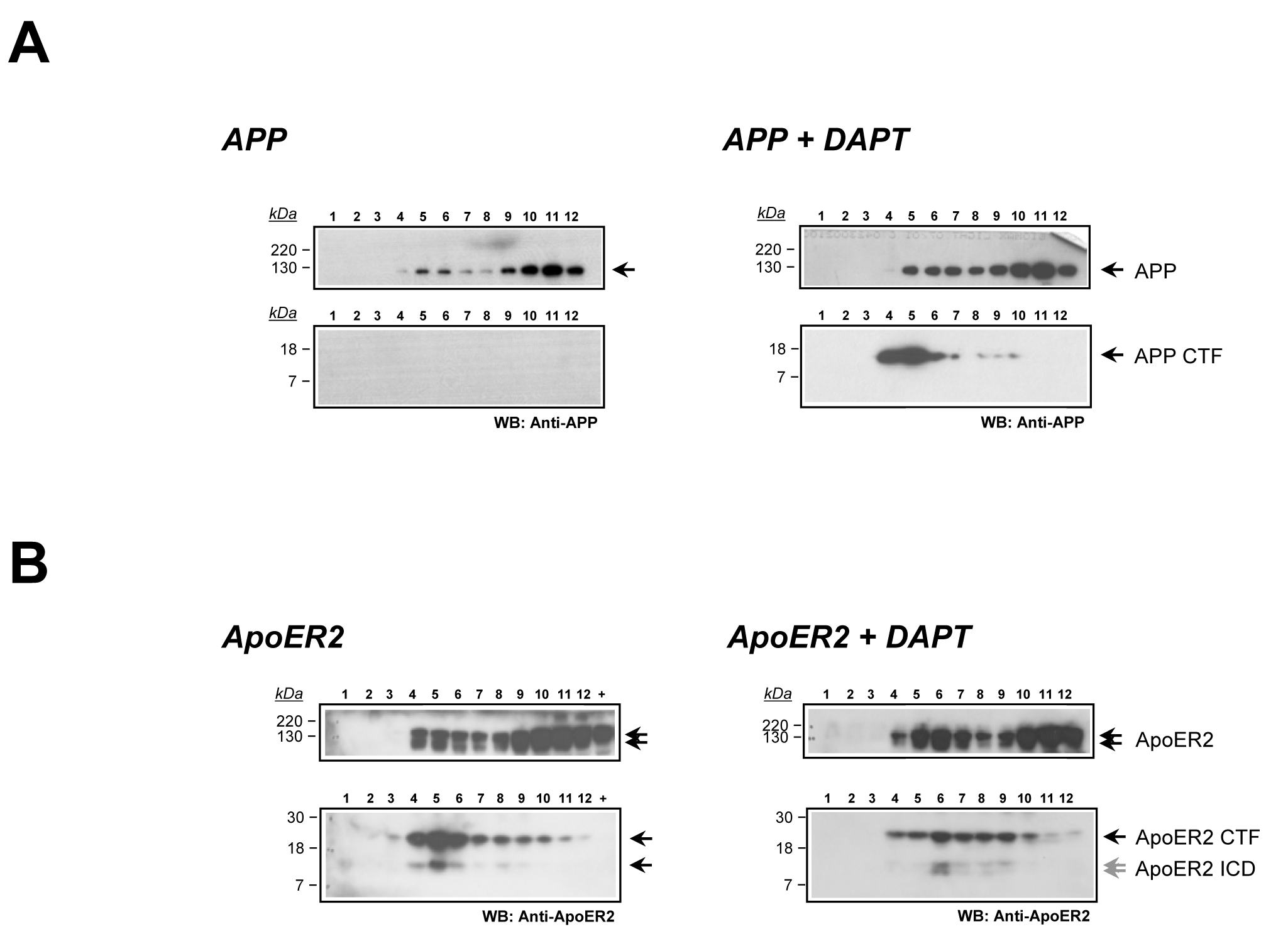

Supplement: Additional file 2 — Figure: Effect of DAPT treatment on APP and ApoER2 CTFs. LRP1-null CHO cells were transiently transfected with plasmids encoding APP695-Myc or ApoER2-HA. After 24 h, cells were incubated with 10 μM DAPT or vehicle (DMSO) for 16 h and lipid rafts were then isolated in a sucrose gradient. After concentration of the sucrose gradients fractions, proteins were subjected to Tris Tricine-PAGE, blotted and probed with anti-APP (A) and anti-ApoER2 (B) antibodies. DAPT treatment improves APP-CTFs detection. However apparently there is still remaining activity that explains the presence of ApoER2-ICDs, resulting from processing of ApoER2-CTFs. [file 1750-1326-2-14-S2.tiff]
